# Utilizing a deep learning model based on BERT for identifying enhancers and their strength

**DOI:** 10.1371/journal.pone.0320085

**Published:** 2025-04-09

**Authors:** Tong Wang, Mengqi Gao

**Affiliations:** School of Computer and Information Engineering, Shanghai Polytechnic University, Shanghai, China; Dalian Maritime University, China

## Abstract

An enhancer is a specific DNA sequence typically located within a gene at upstream or downstream position and serves as a pivotal element in the regulation of eukaryotic gene transcription. Therefore, the recognition of enhancers is highly significant for comprehending gene expression regulatory systems. While some useful predictive models have been proposed, there are still deficiencies in these models. To address current limitations, we propose a model, DNABERT2-Enhancer, based on transformer architecture and deep learning, designed for the recognition of enhancers (classified as either enhancer or non-enhancer) and the identification of their activity (strong or weak enhancers). More specifically, DNABERT2-Enhancer is composed of a BERT model for extracting features and a CNN model for enhancers classification. Parameters of the BERT model are initialized by a pre-training DNABERT-2 language model. The enhancer recognition task is then fine-tuned through transfer learning to convert the original sequence into feature vectors. Subsequently, the CNN network is employed to learn the feature vector generated by BERT and produce the prediction results. In comparison with existing predictors utilizing the identical dataset, our approach demonstrates superior performance. This suggests that the model will be a useful instrument for academic research on the enhancer recognition.

## Introduction

Enhancers, which are located in close proximity to structural genes, belong to a type of end-cis-acting DNA sequence regulatory components. Enhancers have a crucial impact on in the regulation of gene expression across various cell lines and at various temporal stages. During the development of eukaryotic cells, enhancers exert their influence on promoters by binding to transcription factors (TF), cofactors, and chromatin compound in order to enhance the transcriptional activity of promoters. This ultimately results in a growth on the frequency of gene transcription. Enhancers and their corresponding gene promoters are in close physical proximity to each other through the formation of chromatin loops. This precise arrangement allows for the spatio-temporal specific expression of genes to be regulated effectively [1,2]. Mutations in enhancers are closely associated with diseases. Some research has indicated that mutations in enhancers can result in alterations in transcription factor binding sites (TFBS), impacting the binding of transcription factors and chromatin [1,3]. Consequently, these changes can contribute to the growth of certain diseases [3]. In addition, enhancers consist of various subtypes, including weak enhancers and strong enhancers. Therefore, accurately identifying the presence of enhancers and their strength is essential for disease therapies and drug targeting purposes. However, the identification of enhancers poses a significant challenge due to their distribution in non-coding regions of the genome, lack of special sequence characteristics, and distance from the target promoter.

The earliest predictions of enhancers relied on traditional biological methods. Some studies have utilized conserved sequence and TFBS data to recognize enhancers [4–7], while others have employed transcription factor binding data, containing and ChIP-seq data of the transcription coactivator P300 and ChIP-seq data of transcription factors for the enhancer identification [8–11]. Additionally, histone revision data [12,13] and eRNA data [14–18] can also be utilized for the identification of enhancers. This method is capable of obtaining accurate the enhancer information; however, it is time-consuming and expensive with low operability. With the advancement of high-speed sequencing technology of the whole genome, the rapid increase in an enhancer sequence data provides a wealth of training data for predicting enhancers, as well as facilitating the design and implementation of prediction tools. For example, some machine learning methods are used to predict enhancers.

There are numerous techniques available for predicting enhancers using traditional machine learning methods. Liu et al. have proposed a two-layer predictive model, iEnhancer-2L [19], which is capable of recognizing enhancers and their respective types. iEnhancer-2L first determines whether a given sequence is an enhancer and then recognizes the class of the enhancer. Based on this, the research team continued to enhance the model and developed iEnhancer-EL [20] which is an improved version of the iEnhancer-2L released in 2018. Similarly, the EnhancerPred [21] predictor developed by Jia et al. also predicts enhancers and their strengths in a two-layer model. In contrast to iEnhancer-2L, this approach combined three feature encodings to generate hybrid features. To complement the conventional features used in previous methods, Le et al. used word embeddings as inputs and trained SVM algorithms for the development of the iEnhancer-5step in 2019 [22]. Khan et al. developed a prediction tool piEnPred [23] in 2021. piEnPred employed a SVM classifier and optimal hybrid features (a combination of CKSNAP, DCC, PseDNC, and PseKNC). In 2021, Cai et al. proposed “XG-Boost” as the foundational classifier for constructing a two-layer predictor known as iEnhancer-XG [24]. Niu et al. employed iEnhancer-EBLSTM [25] in 2021. The DNA sequence was transformed into a digital sequence and an ensemble model was developed to recognize enhancers based on the BLSTM algorithm. Additionally, iEnhancer-RF [26] was put forward based on a random forest method by Lim et al. in 2021.

Deep learning techniques have evolved from traditional artificial neural network frameworks and have shown significant improvements in prediction performance across various research areas. Among the various deep learning approaches, convolutional neural networks (CNN) and recurrent neural networks (RNN) have garnered significant attention and are widely utilized in the prediction of enhancers. In 2019, Nguyen and colleagues utilized CNN to identify enhancers and their respective types, developing a prediction model known as iEnhancer-ECNN [27]. However, it is important to note that CNN is limited in their ability to focus on only partial information. On the other hand, Enhancer-DRRNN [28] employs RNN for the identification of enhancers and their types. Nevertheless, RNN is susceptible to gradient disappearance when handling long sequences.

In the past few years, natural language processing (NLP) technology has experienced rapid progress [29]. DNA is commonly considered as the “language of life,” utilizing an alphabet composed of four nucleic acids. Among these, DNA sequences can be regarded as textual data [30]. Nucleic acids serve as the words in the biological language, while the regulatory functions and structure provide semantic and syntactic information in enhancer sequences. Due to the similarity between DNA sequences and natural speech, NLP methods have been successfully utilized in the enhancer recognition. For instance, Enhancer-MDLF [31]uses nucleic acid embeddings learned from dna2vec to identify enhancers. However, due to dna2vec relying solely on local information about its neighbors, Enhancer-MDLF is unable to capture global context relationships in enhancer sequences.

In order to address this issue, a pre-training language model with attention mechanism from the field of NLP is used to identify enhancers. For instance, BERT-Enhancer [32] integrates the bidirectional encoder representation of transformers (BERT) [33] as an embedding model to transform the original enhancer sequence into a vector representation. Although BERT- Enhancer achieves comparable performance with existing methods, it is a pre-training BERT model used on human language corpus. Therefore, the BERT- Enhancer model does not contain any prior knowledge about biological sequences. To tackle this issue, a pre-training biological language model called DNABERT-2 has been proposed [34]. It is a pre-training model on a comprehensive multi-species genome. However, the primary purpose of these pre-training models is to acquire generalized descriptions of biological sequences rather than for special tasks. Therefore, in order to advance study on enhancer recognition, a BERT-based enhancer language model is employed essentially.

In the present study, a DNABERT2-based transfer learning model, named DNABERT2-Enhancer, which incorporates a unique fine-tuning architecture is introduced. It is composed of a pre-training BERT model and a CNN model. The enhancer prediction model relies exclusively on DNA sequence data. The identification task is completed in two stages. The initial phase involves determining the classification of the sequence to see if it belongs to the category of enhancers. If it does, the second phase entails predicting the strength of the enhancer, distinguishing between strong and weak enhancers. Experiments show that DNABERT2-Enhancer surpasses other methods on standard datasets, suggesting its potential to introduce a novel approach to biological sequence modeling.

## Materials and methods

### Benchmark dataset

In this study, our objective is to identify enhancers (classified as either enhancer or non-enhancer) and determine their activity level (classified as strong or weak). The dataset mentioned in this article is derived from the dataset utilized by Liu in his research [20], as well as by Basith in his study. Liu’s dataset is utilized by other predictors as well such as [22,27]. The enhancer sequences were divided into 200 bp fragments and filtered by CD-HIT [35]. Highly similar samples were removed to ensure that the remaining samples were less than 80% similar. The dataset encompasses 1484 enhancer sequences, categorized into two distinct subsets: 742 sequences identified as strong enhancers and another 742 sequences designated as weak enhancers, as well as an equal number of 1484 non-enhancer sequences. Furthermore, to evaluate the performance of our models, we applied independent datasets made up of 200 enhancer sequences (100 classified as strong and 100 as weak) as well as 200 sequences that were not enhancers.

Table 1 displays a statistical summary for Liu’s dataset for two layers, including information on the sizes of the training set and the independent datasets.

**Table 1:** Statistical summary of training and testing subsets of Liu’s datasets.

Layer	Training		Testing	
Positives	Negatives	Positives	Negatives
First layer	1484	1484	200	200
Second layer	742	742	100	100

The Basith’s dataset is a more comprehensive dataset that consists of eight subsets. Each subset contains sequences derived from a specific cell line, including GM12878, HMEC, HEK293, HUVEC, HSSM, K562, NHEK and NHLF. Unlike the Liu’s dataset with fixed sequence lengths, the sequence lengths in the Basith’s dataset are variable, ranging from 204 to 2000 base pairs (bp). Redundancy was reduced to 60% for each cell line using CD-HIT. Additionally, this benchmark dataset has an equal number of enhancer and non-enhancer sequences in the training set, but in the test set, the number of non-enhancer sequences is more than twice that of enhancer sequences. Therefore, the Basith’s dataset is closer to real-world scenarios. Indeed, despite its comprehensive nature, the Basith’s dataset also has its limitations. The Basith’s dataset solely comprises enhancer and non-enhancer sequences, lacking data on strong and weak enhancers. A statistical summary of Basith’s dataset for each cell type is shown in Table 2 with information about the size of training and independent datasets.

**Table 2:** Statistical summary of training and testing subsets of Basith’s datasets for different cell types.

Cell lines	Training		Testing	
	Positives	Negatives	Positives	Negatives
GM12878	2187	2187	1187	2356
HMEC	3333	3333	1795	3590
HEK293	3756	3756	2662	5324
HUVEC	4750	4750	2559	5118
HSMM	2821	2821	1520	3040
K652	3318	3318	1787	3754
NHEK	2896	2896	1559	3118
NHLF	1462	1462	788	1576

This study involves two distinct layers for Liu’s dataset. The first layer focuses on identifying the presence of an enhancer versus a non-enhancer (layer 1), serving as the foundation for further analysis. Subsequently, the second layer differentiates between strong enhancers and weak enhancers (layer 2). In the first layer, the presence of an enhancer is considered as a positive instance. In the second layer, strong enhancers are treated as positive samples, while weak enhancers are deemed as negative samples. Conversely, for Basith’s dataset, the model only encompasses a single layer, which distinguishes between enhancers and non-enhancers.

### DNABERT2-enhancer model

**Fig 1:**
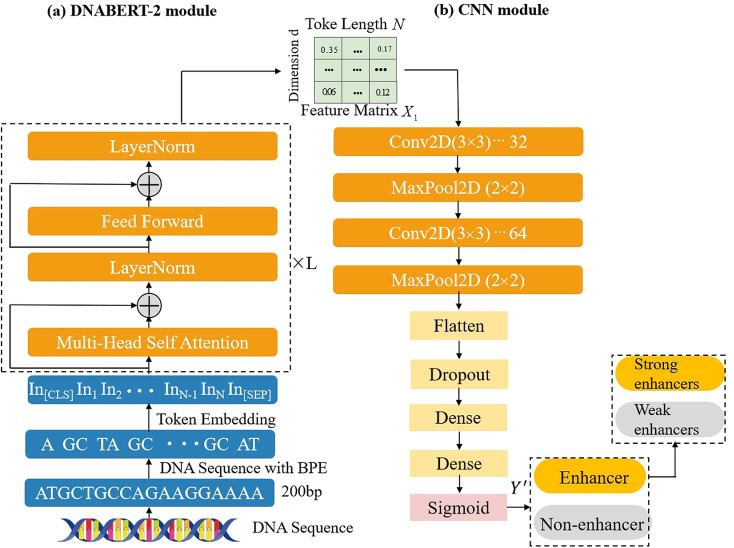
The architecture of DNABERT2-enhancer includes two models: (A) DNABERT-2 model (B) CNN model.

The general structure of the DNABERT2-Enhancer is illustrated in Fig 1. It includes two models: the pre-training DNABERT-2 model and the CNN model. DNABERT-2 is a BERT model pre-training specifically for encoding DNA sequences, effectively capturing intricate long-range dependencies in these sequences. Instead of directly utilizing the outputs of DNABERT-2, the CNN model employs convolutional neural networks (CNNs) to extract meaningful feature vectors from the representations generated by the BERT model. These feature vectors are then transformed into a flattened format and subsequently input into a multi-layered feed-forward neural network, which serves the purpose of classification. A detailed description of our model follows below.

### DNABERT-2 model

DNABERT-2 is the 2.0 version of DNABERT. DNABERT was the first DNA language model based on BERT. It underwent rigorous pre-training using the vast dataset of the entire human genome [36]. It reveals the human genome from a linguistic perspective. Despite its widespread application, the initial implementation of DNABERT still had some technical limitations. Specifically, it had three shortcomings: first, pre-training was only done on the human genome, disregarding sequence diversity and conservation between species. Second, the utilization of k-mers for tokenization led to unintended information disclosure and a general decline in computational efficiency during pre-training, which hindered its scalability. Finally, there were deficiencies in both efficiency and effectiveness in handling long input sequences. These shortcomings highlight the necessity for further development in the field of DNA language models. DNABERT-2 has made improvements in these three shortcomings. Firstly, it undergoes pre-training on a comprehensive multi-species genome, rather than solely focusing on the human genome. Secondly, byte pair encoding (BPE) is utilized to replace k-mer tokenization. BPE is a widely used data compression algorithm for large language models [37]. Tokens are constructed by iteratively incorporating the most commonly occurring genomic segments in the corpus. BPE effectively overcomes the limitations of k-mer tokenization. Lastly, DNABERT-2 eliminates the input length limit by replacing the original positional embedding with an attention with linear bias (ALiBi) [38].

The BERT model is comprised of two separate components: the module responsible for pre-processing BERT inputs and the pre-training BERT module. In the BERT input pre-processing module, DNABERT-2 utilizes BPE [37] to tokenization DNA sequences. BPE is a compression algorithm that has been employed in natural language processing area as a word segmentation strategy widely. For more detailed information on BPE, please refer to [34] which provides a comprehensive analysis and in-depth discussion of BPE technology.

Then, two unique tokens, namely [CLS] introduced at the start and [SEP] appended at the termination of the tokenized sequence, are inserted individually. Each token is then put into the embedding module then converted into a vector. In the DNABERT model, there are not only token embeddings, but also position embeddings. DNABERT-2 model adopts the approach of Attention with ALiBi [38]. Instead of adding location embeddings to the input, a non-learned biases and fixed set of static are added to each attention computation in order to combine positional information to the attention score.

DNABERT-2 employs the transformer encoder architecture as the backbone of its pre-training BERT module. The feature matrix $X_{1}$ is then obtained by *L* cascade encoders. Each encoder consists of a multi-headed self-attention unit, a feed-forward neural network component, and dual normalization layers. In the *i* -th encoder, the multi-head self-attention can be obtained as follows:


$$Multihead(X^{i})=Concat(head_{1}^{i},head_{2}^{i},...,head_{n}^{i})W^{O,i}$$ (1)

For the *i* -th encoder, the input matrix $X^{i}$ is processed by *n* self-attention heads. The outputs of these heads are then transformed by the output transformation matrix $W^{O,i}$, with each $head^{i}$ computation detailed in the following:


$$head^{i}=soft\max(\frac{W^{Q,i}X^{i}{\left(W^{K,i}X^{i}\right)}^{T}}{\sqrt{d_{k}}})W^{V,i}X^{i}$$ (2)

$W^{Q,i}$, $W^{K,i}$ and $W^{V,i}$ are the transformation matrices for the query, key, and value components of a head, respectively. $d_{k}$ represents the dimension count of the key matrix.

The resultant output of the multi-head attention $MultiHead(X^{i})$ is then connected within the residual of the input $X^{i}$ as inputs to the normalized layer. Please use the following formula for calculation:


$$Y^{i}=LayerNorm(MultiHead(X^{i})+X^{i})$$ (3)

Subsequently, the normalized results are fed into the feed-forward neural network, and the calculation formula is as follows:


$$FFN(Y^{i})=\max(0,Y^{i}W_{1}+b_{1})W_{2}+b_{2}$$ (4)

where $W_{1}$, $W_{2}$, $b_{1}$ and $b_{2}$ are the trainable weight parameters within the feed-forward layer.

The output of the *i* -th encoder is achieved by normalizing the residual connection between $Y^{i}$ and $FFN(Y^{i})$, and the calculation formula is as follows:


$$X^{i+1}=LayerNorm(Y^{i}+FFN(Y^{i}))$$ (5)

In the end, the output of DNABERT-2 can be obtained through the cascade *L* encoder as follows:


$$X_{1}=X^{i+1}\in R^{d\times N}$$ (6)

In which, *d* denotes the dimensionality of the word vector, while *N* denotes the total quantity of tokens.

The DNABERT-2 utilizes a BERT model architecture characterized by (*L* = 12, *H* = 768, *A* = 12). *L* defines the total number of layers in the model, specifically, there are 12 Transformer units. *H* represents the size of the hidden layer, with each token being a 768-dimensional vector. *A* represents the self-attention head, and there are a total of 12 attention heads in the model. In this research, we consider enhancer DNA sequences as “sentences in natural language” and convert them into fixed-length feature matrices using the BERT model.

### CNN model

In the CNN model stage, high-level local features from the feature matrix $X_{1}$ is obtained by a succession of convolutional layers [39]. The CNN framework is composed of two key parts: a convolutional sub-module responsible for convolutional operations, and a classification sub-module tasked with categorizing inputs. The convolutional network modules utilize convolutional layers and maximum pooling layers to learn higher-order representations of features, which are expressed as:


X1'=ReLU(Conv(X1)) (7)

where Conv(·) represents the convolution operation. Following this, the max-pooling layer is obtained in the following:


$$P=MaxPooling\left(X_{1}^{’}\right)$$ (8)

where *P* represents the result of applying the max-pooling function, MaxPooling(·) denotes the max-pooling operation.

The eigenvector matrix is processed through a series of perceptron layers, ultimately yielding a predictive output $Y^{'}$ that represents the probability of classifying the input DNA sequence as an enhancer. This prediction is facilitated by a multi-layer perceptron comprising two fully connected layers, with the first layer incorporating dropout for regularization and dropout(·) the second layer utilizing a sigmoid activation function for the final classification decision. In essence, the above processes can be summarized as:


$$Y^{'}=sigmoid(MLP(MLP(dropout(flatten(P_{1})))))$$ (9)

where $Y^{'}$ represents the predicted value. sigmoid(·) denotes the sigmoid function. MLP(·) denotes feed-forward neural networks. denotes the dropout operation. flatten(·) defines the flatten operation.

## Results

### Performance evaluation metrics

In this article, we utilize several metrics [40] to measure the performance of our model, containing specificity (SP), sensitivity (SN), accuracy (ACC) [41], balanced accuracy (BACC), Matthew’s correlation coefficient (MCC) and Area Under Curve(AUC). SP is defined as the percentage of all instances in which the model accurately classifies as negative. SN is defined as the percentage of actual positive cases that the model correctly identifies as positive. ACC signifies the overall correctness of a model’s predictions, constituting a fundamental benchmark for assessing its performance. It is determined as the proportion of accurately forecasted examples among all examples that were predicted. Compared with ACC, BACC is more suitable to evaluate performance on an imbalanced dataset. Since the Liu’s dataset used in this study is balanced, we use ACC to evaluate the model’s performance. However, since the Basith’s dataset is imbalanced, we use the BACC metric instead of ACC to assess the model’s performance. MCC is a comprehensive metric that assesses the overall quality of a classification model’s predictions by examining its performance in each of the four quadrants of the confusion matrix. A superior score reflects balanced excellence across true positives (TP), true negatives (TN), false negatives (FN), and false positives (FP). TP represents the count of correctly identified positive samples. TN represents the number of correctly classified negative samples. FN represents the number of instances where the model failed to detect the presence of a positive class. FP represents the count of incorrectly identified positive samples. The MCC considers the number of true negatives samples, true positives samples, false negatives samples and false positives samples, serving as a balancing indicator. The values range from -1 to + 1: -1 indicates complete inconsistency, 0 denotes random prediction, and + 1 signifies complete consistency. Receiver Operation Characteristics Curve-Area Under Curve (ROC-AUC) [42] is the most crucial experimental evaluation index in this study, as it reflects the most comprehensive prediction performance. AUC represents the area under the ROC curve and the horizontal axis, and its value cannot exceed 1 [43]. The ROC curve naturally resides above the $SN=\frac{TP}{TP+FN}$ line, featuring an AUC range from 0.5 to 1. The closer the AUC approaches 1.0, the stronger the verification of the detection method’s genuineness and predictive power. Conversely, an AUC score of 0.5 signifies a diminished authenticity and practical irrelevance for the given application [44].

The detailed equation used for the calculation is as indicated below: The detailed equation used for the calculation is as indicated below:


$$SN=\frac{TP}{TP+FN}$$ (10)

$$SP=\frac{TN}{TN+FP}$$ (11)

$$ACC=\frac{TP+TN}{TP+TN+FN+FP}$$ (12)

$$BACC=\frac{SN+SP}{2}$$ (13)

$$MCC=\frac{TP\times TN-FP\times FN}{\sqrt{\left(TP+FP)(TP+FN)(TN+FP)(TN+FN\right)}}$$ (14)

In the subsequent study, we will comprehensively evaluate the predictive performance of the trained DNABERT2-Enhancer and other relevant models by combining the 6 indicators (SP, SN, MCC, ACC, BACC and AUC).

## Results

In our experiments, for the DNABERT2-Enhancer model, we set 100 training epochs, with a learning rate of 0.001 and a batch size of 16. To further enhance the model’s generalization ability and prevent overfitting, we introduced a dropout layer with a probability of 0.6. This layer randomly drops certain neural network units during the training phase, contributing to the model’s overall robustness and preventing excessive adaptation to the training data.

**Fig 2:**
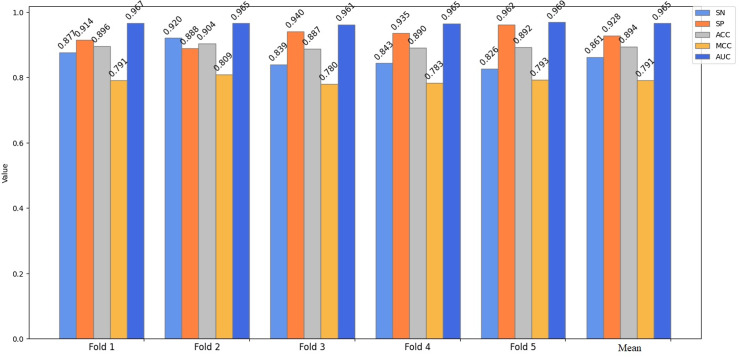
The 5-fold cross-validation results obtained by the DNABERT2-enhancer on Liu’s training dataset (layer 1).

**Fig 3:**
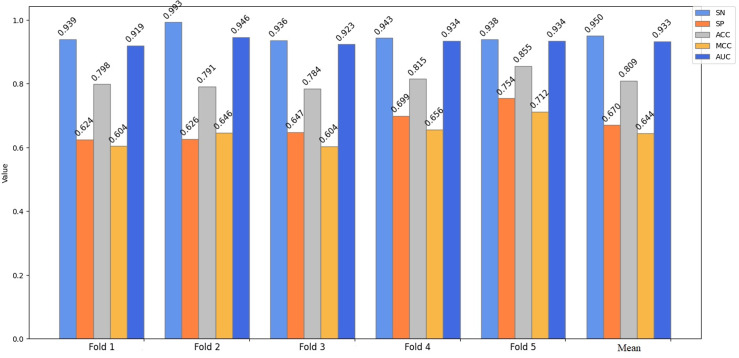
The 5-fold cross-validation results obtained by the DNABERT2-enhancer on Liu’s training dataset (layer 2).

For performance evaluation, a cross-validation technique is adopted, involving random division of the complete dataset into k separate parts. During the process, k-1 of these parts are dedicated to model training, while the remaining 1 part is the testing dataset. In the course of our experiment, we have set the value of k to 5. In the end, the mean performance index of k validations was calculated as the model’s performance measure. Fig 2 presents the outcomes of a 5-fold cross-validation for the first phases of the model on Liu’s training dataset. In the cross-validation test, the proposed predictor obtained an average prediction result of SN of 86.1%, SP of 92.8%, ACC of 89.4%, MCC of 0.791 and AUC of 0.965. In the second stage, the proposed predictor obtained an average prediction result of SN of 95.0%, SP of 67.0%, ACC of 80.9%, MCC of 0.644 and AUC of 0.933, as illustrated in Fig 3. The outcomes of the first stage surpass those of the second stage on Liu’s training dataset. This is due to the fact that the disparity between an enhancer and a non-enhancer is more pronounced than that between a strong enhancer and a weak enhancer. The greater the discrepancy, the easier it becomes to discern. It is evident that the values of SP, SN, MCC, ACC, AUC and mean indexes on the training data set have relatively small fluctuations and are relatively stable, effectively avoiding the over fitting problem. Therefore, this indicates that the DNABERT2-Enhancer model had a good performance on Liu’s training dataset.

Table 3 displays the performance of five-fold cross-validation results on the eight cell lines of the Basith’s dataset.

**Table 3:** The 5-fold cross-validation results obtained by the DNABERT2-Enhancer on Basith’s training dataset (layer 1).

Cell line	ACC	AUC	SN	SP	MCC
GM12878	**0.926**	**0.978**	**0.859**	**0.967**	**0.772**
HMEC	**0.843**	**0.906**	**0.838**	**0.861**	**0.663**
HEK293	**0.889**	**0.952**	**0.851**	**0.933**	**0.764**
HUVEC	**0.804**	**0.876**	**0.782**	**0.837**	**0.609**
HSMM	**0.775**	**0.859**	**0.765**	**0.788**	**0.586**
K652	**0.851**	**0.917**	**0.842**	**0.876**	**0.671**
NHEK	**0.822**	**0.881**	**0.809**	**0.934**	**0.750**
NHLF	**0.857**	**0.918**	**0.836**	**0.855**	**0.685**

**Fig 4:**
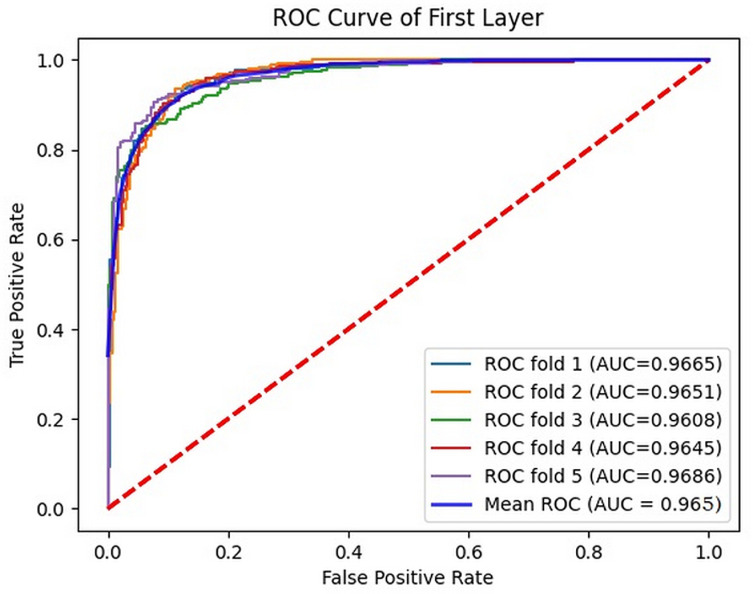
The ROC curves obtained by the DNABERT2-enhancer on Liu’s training dataset (layer 1).

**Fig 5:**
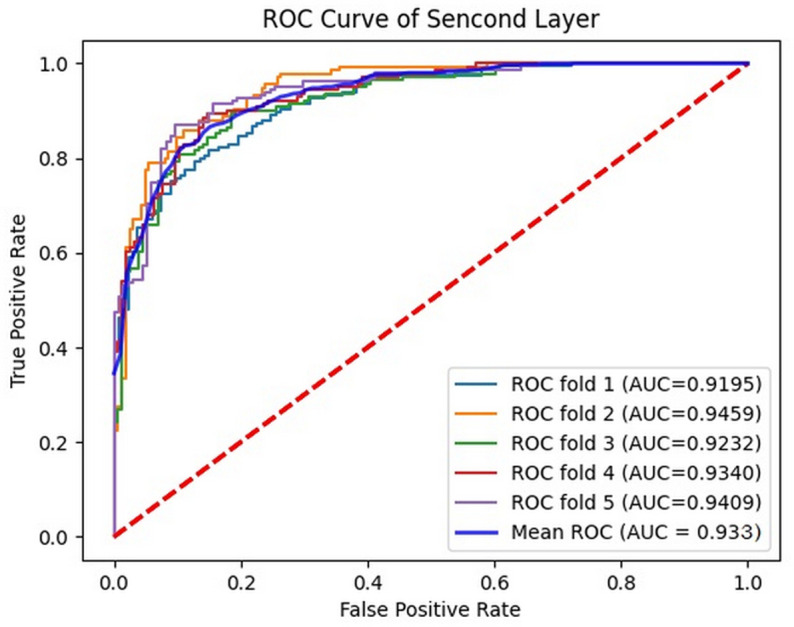
The ROC curves obtained by the DNABERT2-enhancer on Liu’s training dataset (layer 2).

The ROC curve of the DNABERT2-Enhancer model on the training dataset is shown in Figs 4 and 5. In the first layer (enhancer identification), Fig 4 illustrates the ROC curve for 5-fold cross-validation, yielding an average AUC of 0.965, while in the second layer (enhancer strength prediction), Fig 5 displays the ROC curve for 5-fold cross-validation with an average AUC of 0.933. These results illustrate that the introduced DNABERT2-Enhancer model exhibits good stability.

### Comparison of the proposed model with existing methods

Table 4 presents the 5-fold cross-validation performance of the DNABERT2-Enhancer model in comparison with three existing prediction methods. Five metrics were compared using the same benchmark dataset, including ACC, AUC, SN, SP, and MCC. ACC serves as an indicator of the predictor’s overall precision, while MCC and AUC provide metrics for assessing the model’s robustness and comprehensive performance in real-world applications. Additionally, SN and SP measure the predictor from two different perspectives, which in fact complement each other. As depicted in Table 4, in the classification of enhancers and non-enhancers (first layer) and the prediction of enhancer strength (second layer), the proposed method demonstrates excellent performance in the five metrics on Liu’s training set.

**Table 4:** Performance comparison between DNABERT2-Enhancer and other methods by the 5-fold cross-validation results on liu’s training dataset.

Layer	Method	ACC	AUC	SN	SP	MCC	Source
First layer	iEnhancer-ECNN	0.769	0.832	0.785	0.752	0.537	[27]
	BERT-Enhancer	0.762	-^2^	0.795	0.730	0.525	[32]
	iEnhancer-BERT	0.794	0.876	–	–	0.593	[45]
	DNABERT2-Enhancer	**0.894** ^1^	**0.965**	**0.861**	**0.928**	**0.791**	This study
Second layer	iEnhancer-ECNN	0.678	0.748	0.791	0.564	0.368	[27]
	BERT-Enhancer	–	–	–	–	–	[32]
	iEnhancer-BERT	0.653	0.703	–	–	0.310	[45]
	DNABERT2-Enhancer	**0.809**	**0.933**	**0.950**	**0.670**	**0.644**	This study

^1^The entries are bolded to highlight the best results under equivalence experiments.

^2^“-” indicates that the metrics value is not provided.

In this research, the introduced model is contrasted with several other enhancer identification models utilizing the test dataset. These models include EnhancerPred [21], iEnhancer-EL [20], iEnhancer-ECNN [27], BERT-Enhancer [32], iEnhancer-XG [24] and iEnhancer-BERT [45]. EnhancerPred [21] employs a two-layer SVM-based classifier system. The first layer discriminates between enhancer and non-enhancer sequences, while the second layer categorizes the strength or intensity of enhancer sequences. This predictive model incorporates three distinct encoding methodologies for its construction. iEnhancer-EL [20] utilizes the concept of ensemble learning to devise a two-layer ensemble classifier, which integrates multiple prediction strategies to enhance accuracy. iEnhancer-ECNN [27] incorporates deep learning methodologies into enhancer identification. BERT-Enhancer [32] constructs 2D-CNN architectures by utilizing the sequence representations derived from pre-training BERT models, leveraging their contextual understanding of genomic sequences. iEnhancer-XG [24] is a two-stage enhancer recognition model that utilizes XG-Boost, a powerful boosting algorithm, in conjunction with five fundamental physicochemical properties to make predictions. iEnahncer-BERT [45] develops 2D-CNN networks by exploiting the sequence embeddings generated by pre-training DNABERT models, integrating the advanced language modeling capabilities of BERT into enhancer prediction. In 2024, the Enhancer-MDLF [31], a Multi-input Deep Learning Framework is designed to identify enhancers. This approach amalgamates word vector features derived from the human genome sequence and motif features extracted from the position weight matrix(PWM) of motifs.

The outcomes of the comparative analysis are given in Table 5. DNABERT2-Enhancer outperformed other predictors in all five measures. In the first layer (enhancer recognition), DNABERT2-Enhancer achieved optimal performance indicators of 82% (ACC), 0.868 (AUC), 81.5% (SN), 77.5% (SP) and 59.1 (MCC). The ACC and AUC of DNABERT2-Enhancer were 1.75-7.2% and 2.4-5.1% higher than other predictors respectively. In the second layer (enhancer classification), DNABERT2-Enhancer obtained optimal performance indicators: ACC (75%), AUC (0.821), SN (89%), SP (67%) and MCC (0.544). The ACC, AUC and MCC of DNABERT2-Enhancer were higher by 4.9-14%, 0.9-14.1%, and 13.6%-32.2% compared to other predictors respectively. These results indicate that DNABERT2-Enhancer demonstrates superior performance in enhancer recognition and classification compared to existing predictors.

**Table 5:** Performance comparison between DNABERT2-Enhancer and other methods on Liu’s testing dataset.

Layer	Method	ACC	AUC	SN	SP	MCC	Source
First layer	EnhancerPred	0.774	-^2^	0.7197	**0.8282**	0.55	[21]
	iEnhancer-EL	0.748	0.817	0.710	0.785	0.496	[20]
	iEnhancer-ECNN	0.765	0.834	0.790	0.740	0.531	[27]
	BERT-Enhancer	0.756	–	0.80	0.712	0.514	[32]
	iEnhancer-XG	0.7575	–	0.7400	0.7750	0.515	[24]
	iEnhancer-BERT	0.793	0.844	–	–	0.585	[45]
	Enhancer-MDLF	0.8025	–	**0.84**	0.765	**0.6067**	[31]
	DNABERT2-Enhancer	**0.820** ^1^	**0.868**	0.815	0.775	0.591	This study
Second layer	EnhancerPred	0.682	–	0.7116	0.6523	0.36	[21]
	iEnhancer-EL	0.610	0.680	0.540	0.650	0.222	[20]
	iEnhancer-ECNN	0.695	0.759	0.840	0.550	0.408	[27]
	BERT-Enhancer	–	–	–	–	–	[32]
	iEnhancer-XG	0.635	–	0.7000	0.5700	0.272	[24]
	iEnhancer-BERT	0.701	0.812	–	–	0.401	[45]
	Enhancer-MDLF	–	–	–	–	–	[31]
	DNABERT2-Enhancer	**0.750**	**0.821**	**0.890**	**0.670**	**0.544**	This study

^1^The entries are bolded to highlight the best results under equivalence experiments.

^2^“-” indicates that the metrics value is not provided.

To thoroughly evaluate the capability of DNABERT2-Enhancer in predicting cell-specific enhancers, we conducted a detailed comparison with the Enhancer-IF model, which is specifically designed for predicting cell-specific enhancers. As demonstrated in Table 6, DNABERT2-Enhancer outperformed Enhancer-IF across all five evaluation metrics and in all tested cell lines, fully demonstrating its significant superiority.

**Table 6:** Performance comparison of DNABERT2-Enhancer and Enhancer-IF on Basith’s testing datasets for eight cell types.

Cell line	Method	BACC	AUC	SN	SP	MCC	Source
GM12878	Enhancer-IF	0.814	0.901	0.755	0.873	0.626	
	DNABERT2-Enhancer	**0.885**	**0.952**	**0.833**	**0.937**	**0.716**	This study
HMEC	Enhancer-IF	0.753	0.839	0.740	0.765	0.485	
	DNABERT2-Enhancer	**0.816**	**0.874**	**0.804**	**0.828**	**0.612**	This study
HEK293	Enhancer-IF	0.817	0.893	0.775	0.858	0.625	
	DNABERT2-Enhancer	**0.861**	**0.935**	**0.820**	**0.902**	**0.721**	This study
HUVEC	Enhancer-IF	0.737	0.811	0.752	0.722	0.450	
	DNABERT2-Enhancer	**0.779**	**0.852**	**0.753**	**0.805**	**0.573**	This study
HSMM	Enhancer-IF	0.718	0.794	0.709	0.727	0.417	
	DNABERT2-Enhancer	**0.752**	**0.836**	**0.746**	**0.758**	**0.567**	This study
K562	Enhancer-IF	0.773	0.852	0.773	0.772	0.523	
	DNABERT2-Enhancer	**0.824**	**0.897**	**0.811**	**0.837**	**0.654**	This study
NHEK	Enhancer-IF	0.748	0.826	0.734	0.762	0.477	
	DNABERT2-Enhancer	**0.808**	**0.868**	**0.797**	**0.918**	**0.602**	This study
NHLF	Enhancer-IF	0.794	0.869	0.789	0.798	0.565	
	DNABERT2-Enhancer	**0.832**	**0.903**	**0.821**	**0.843**	**0.672**	This study

The comparison between MCC value and ACC value indicates a greater increase in MCC value. The MCC comprehensively considers the four indexes in the confusion matrix. This balanced index provides higher insight into the evaluation of a model’s performance. It can be concluded that our proposed predictor demonstrates higher stability and overall performance compared to other models. Furthermore, the SN and SP metrics demonstrate remarkable advantages in both the initial and subsequent stages, implying that the proposed model possesses a more balanced and stable performance, excelling in distinguishing between positive and negative samples.

### Interpretability analysis of DNABERT2-Enhancer

We intend to leverage the SHAP (Shapley Additive exPlanations) algorithm alongside t-SNE (t-Distributed Stochastic Neighbor Embedding) technology to conduct an in-depth analysis of the interpretability of the DNABERT2-Enhancer model when integrating DNABERT2 with CNN.

**Fig 6:**
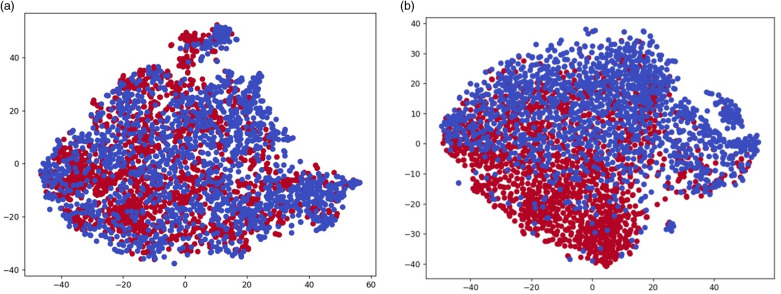
The t-SNE visualization of the feature space distribution. (A) The t-SNE result of the original features. (B) The result of projecting the high-dimensional feature space learned by the DNABERT2-enhancer model into a two-dimensional view.

Specifically, we will utilize t-SNE technology to project each feature vector onto a two-dimensional view, enabling a visual representation of the distribution of enhancers and non-enhancers in the visualization chart. Fig 6 displays the arrangement of enhancers and non-enhancers in the two-dimensional space. The blue dots represent non-enhancers, and the red dots represent enhancers. The first subfigure represents the t-SNE result of the original features, which can be interpreted as the entire sample points failing to exhibit any representative clusters. The features are overlapping in distribution, and there is no clear separation between enhancers and non-enhancers (Fig 6A). The second subfigure shows the result of projecting the high-dimensional feature space learned by the DNABERT2-Enhancer model into a two-dimensional view, where the features exhibit a regular distribution. The degree of separation in the feature space is significantly improved, with reduced overlap (Fig 6B), thereby enhancing performance. This allows us to capture the overall differences between enhancers and non-enhancers. In summary, our method can learn better model decision boundaries. Through this visualization technique, we can gain a more intuitive understanding of the impact of features on model predictions, further deepening our exploration of the model’s interpretability.

**Fig 7:**
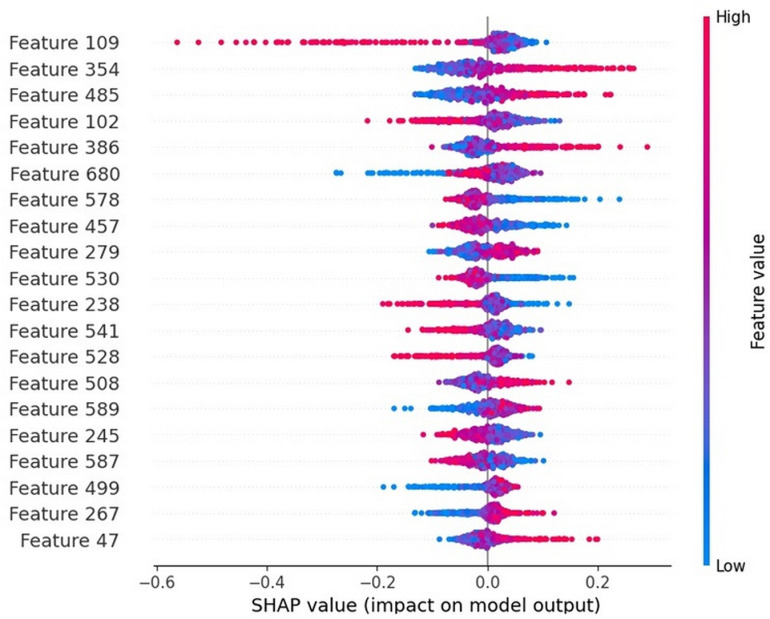
The influence of each of the top 20 features on the recognition of different enhancers.

Simultaneously, we will employ the SHAP algorithm to quantify the contribution of each feature to the prediction results of the DNABERT2-Enhancer model, thereby identifying which features play crucial roles in identifying and classifying enhancers. This will aid us in gaining a deeper understanding of the model’s decision logic and improving its interpretability. Fig 7 reflects the influence of each of the top 20 features on the recognition of different DNA enhancer sequences, where red indicates a positive effect, increasing the likelihood of a sequence being predicted as an enhancer, and blue indicates a negative effect, increasing the likelihood of a sequence being predicted as a non-enhancer. We observe that different features may contribute differently to the final output. Taking Feature 109 in Fig 7 as an example, higher feature values are mainly concentrated in the region where SHAP values are greater than 0, suggesting that Feature has a positive impact on the model’s output. Subsequently, combining known biological knowledge, we can further explore how these key features relate to enhancer activity, thereby providing insights into biological mechanisms.

### The DNABERT2-Enhancer web server

To make it more convenient for users to access and utilize the DNABERT2-Enhancer model, we have developed a web server and successfully deployed it online. Now, users can directly use our model for enhancer prediction and analysis through online access. The web server can be accessed at the following address:DNABERT2Enhaner.dongtaiyuming.net. On this server, users can input DNA sequences in two ways: either by directly pasting FASTA-formatted DNA sequences into a text box, or by uploading a file containing FASTA-formatted DNA sequences through a file selection dialog. After clicking the “Submit Sequence” button, users can obtain the corresponding processing results. Additionally, you can download the source code and data from the DNABERT2-Enhancer web server at DNABERT2Enhancer.dongtaiyuming.net.

## Discussion

Enhancers play a pivotal role in various cellular processes and the pathogenesis of diseases. The accurate identification of enhancers holds significant importance in understanding the cellular processes and other potential functional mechanisms. Over the past decade, machine learning algorithm has been utilized to identify the types of enhancers. While some computational methods have been proposed, the current algorithms for predicting enhancers lack sophistication in encoding DNA sequence information. This simplistic approach results in a limited capacity to learn and capture the intricate features of DNA sequences. To enhance the coding performance of DNA recombinant sequences, we aim to capture the implicit information within them. In this study, we employ the language model DNABERT-2 from the field of natural language processing to effectively model DNA sequences. DNABERT-2 effectively captures sequence properties in DNA sequences within unlabeled big data. The DNA sequence is represented as a continuous word vector. The DNA sequences were transformed into vectors by training DNABERT-2 model. Subsequently, the extracted features are learned through CNN, and the prediction results are generated.

We have conducted a comparison and analysis of the performance of DNABERT2-Enhancer with other predictors, and the results demonstrated DNABERT2-Enhancer obtained the best performance compared to the comparison models. Our models outperformed the existing models for Acc, AUC, SN, SP, and MCC, suggesting that the DNABERT2-Enhancer is a robust and reliable predictor.

Regarding the potential applications of the DNABERT2-Enhancer model, we acknowledge its significant importance in both research and clinical settings. In research, this model can assist researchers in more efficiently identifying and analyzing enhancers, thereby providing deeper insights into the complex mechanisms of gene regulation. This contributes to advancing the field of life sciences and offers new perspectives and clues for the study of related diseases. In clinical contexts, although DNABERT2-Enhancer has not yet been directly applied to clinical diagnosis or treatment, we believe its potential value cannot be overlooked. To more comprehensively present the potential applications of the DNABERT2-Enhancer model, we will enhance the discussion in this area in our subsequent research and attempt to collaborate with clinical experts and medical institutions to explore its feasibility and effectiveness in practical applications.

## Conclusion

In this article, we have employed a computational model called DNABERT2-Enhancer to efficiently differentiate enhancers from non-enhancers based on deep learning. Our proposed model serves two purposes: recognizing enhancers and estimating their strength. The experimental results show that DNABERT2-Enhancer can predict enhancers and their strength accurately. Compared with existing technologies, the model proved to be powerful, valuable, and efficient. In the future, we will investigate feature extraction techniques to enhance the prediction accuracy of the model and enhance its predictive capability.
